# Heterologous Fibrin Biopolymer for Post-Extraction Alveolar Bone Healing in Streptozotocin-Induced Diabetic Rats

**DOI:** 10.3390/jfb17080380

**Published:** 2026-08-03

**Authors:** Suelen Paini, Tania Mary Cestari, Ana Carolina Cestari Bighetti, Rafael Carneiro Ortiz, Rui Seabra Ferreira, Benedito Barraviera, Nathália Dantas Duarte, Rogerio Leone Buchaim, Evelyn Lorene Rodrigues da Silva, Bruna Trazzi Pagani, Gustavo Pompermaier Garlet, Gerson Francisco de Assis, Daniela Vieira Buchaim

**Affiliations:** 1 Department of Biological Sciences, Bauru School of Dentistry (FOB), University of São Paulo (USP), Bauru 17012-901, SP, Brazil; suelenpaini@alumni.usp.br (S.P.); cestari@fob.usp.br (T.M.C.); anacarolinacb@usp.br (A.C.C.B.); rafaelortiz@usp.br (R.C.O.); rogerio@fob.usp.br (R.L.B.); garletgp@usp.br (G.P.G.); gfassis@fob.usp.br (G.F.d.A.); 2 Center for the Study of Venoms and Venomous Animals (CEVAP), Botucatu Medical School (FMB), São Paulo State University (UNESP), Botucatu 18610-307, SP, Brazil; rui.seabra@unesp.br (R.S.F.J.); benedito.barraviera@unesp.br (B.B.); 3 Professional Graduate Program in Clinical Research, Botucatu Medical School (FMB), São Paulo State University (UNESP), Botucatu 18618-687, SP, Brazil; 4 Center for Translational Science and Biopharmaceutical Development (CTS-CEVAP), São Paulo State University (UNESP), Botucatu 18610-307, SP, Brazil; 5 Department of Diagnosis and Surgery, Araçatuba School of Dentistry (FOA), São Paulo State University (UNESP), Araçatuba 16015-050, SP, Brazil; nd.duarte@unesp.br; 6 Postgraduate Program in Comparative and Translational Veterinary Anatomy, Faculty of Veterinary Medicine and Animal Science (FMVZ), University of São Paulo (USP), São Paulo 05508-270, SP, Brazil; 7 Medical School, University Center of Adamantina (FAI), Adamantina 17800-000, SP, Brazil; 83323@fai.com.br; 8 Dentistry School, University of Marilia (UNIMAR), Marilia 17525-902, SP, Brazil; brunatrazzi@usp.br; 9 Postgraduate Department, Dentistry School, Faculty of the Midwest Paulista (FACOP), Piratininga 17499-010, SP, Brazil

**Keywords:** alveolar bone healing, bone repair, diabetic rat model, fibrin tissue adhesive, streptozotocin, tooth extraction

## Abstract

Type 1 diabetes mellitus (DM1) is characterized by autoimmune destruction of pancreatic β-cells, resulting in insulin deficiency and persistent hyperglycemia, which impair bone metabolism and compromise post-extraction alveolar bone healing. Heterologous fibrin biopolymer (HFB) has previously demonstrated biocompatibility, biodegradability, bioactivity, and osteoconductive properties under normoglycemic conditions. Therefore, the study investigated the association of HFB with post-extraction alveolar bone healing in streptozotocin-induced diabetic rats, using the diabetic blood clot as a biological control. Forty-eight adult male Wistar rats were induced to DM1 by a single intraperitoneal injection of streptozotocin (52 mg/kg). Animals were included after confirmation of hyperglycemia (fasting blood glucose ≥ 250 mg/dL) measured seven days after STZ administration: blood clot (BCG; *n* = 24) or HFB (HFBG; *n* = 24). Seven days after DM1 induction, the right maxillary incisor was extracted, and the sockets were filled according to group allocation. Animals were euthanized at 7, 14, and 42 days post-extraction. Fasting blood glucose levels were monitored, and pancreatic insulin immunohistochemistry was performed to characterize the diabetic condition. Alveolar bone healing was assessed by micro-CT, histological (HE), histomorphometric, and picrosirius red analyses. The diabetic phenotype was confirmed by persistent fasting hyperglycemia (260–589 mg/dL) and reduced pancreatic insulin immunostaining in β-cells. At 42 days, bone volume (BV) was 31.86% higher in the HFBG than in the BCG (*p* < 0.05). BCG exhibited higher trabecular number (Tb.N), while HFBG exhibited higher trabecular thickness (Tb.Th) (*p* < 0.05). Histologically, the HFBG shows a more mature, compact, and organized bone structure, whereas the BCG presents bone tissue with a more trabecular and immature architecture. HFBG showed the lowest percentage of thin fibers at the late stage, whereas BCG maintained similar values from 14 to 42 days (*p* < 0.05). These findings suggest that HFB was associated with favorable changes in selected late-stage parameters of post-extraction alveolar bone healing in an STZ-induced diabetic model.

## 1. Introduction

Diabetes mellitus (DM) is a chronic metabolic disease characterized by hyperglycemia resulting from impaired insulin secretion, defective insulin action, or both mechanisms [1]. According to the International Diabetes Federation (2025), an estimated 589 million adults aged 20 to 79 years live with DM. This represents 11.1% of the world’s population in this age group, making it a public health concern [2,3]. Although type 2 diabetes mellitus (DM2) represents the most prevalent form, type 1 diabetes mellitus (DM1) affects approximately 5–10% of diabetic cases and is frequently diagnosed in children and young adults, persisting throughout the individual’s lifetime [2,4].

DM1 results from autoimmune destruction of pancreatic β-cells, leading to severe insulin deficiency and chronic metabolic dysregulation [5]. Insulin has an anabolic effect on bone tissue [6]. Therefore, the absence of insulin compromises bone metabolism, including osteoblastic activity and matrix mineralization, resulting in decreased bone mineral density (BMD) [7,8]. In this context, DM1 is associated with lower BMD than DM2 [9]. This difference may be explained by abnormal bone development in youth with DM1, which may negatively affect BMD later in life [10,11]. Moreover, sustained hyperglycemia interferes with collagen metabolism, contributing to deterioration of bone quality [12].

In Dentistry, the incidence of tooth extraction in individuals with DM is approximately 1.88 times higher than in non-diabetic individuals [13]. In diabetic patients, alveolar bone healing after tooth extraction is frequently impaired, mainly due to delayed wound closure and prolonged inflammatory response [14]. Young adults with DM1 exhibit significantly more alveolar bone loss (24.6–43.9%) than non-diabetic individuals (5–10%) [15]. Therefore, several therapeutic strategies have been investigated to improve post-extraction alveolar bone healing under DM conditions, including photobiomodulation [16], bioactive compounds such as ellagic acid and curcumin [17,18], growth factors [19,20], and loaded hydrogels [21,22].

Despite these advances, evidence for effective biomaterial-based strategies to optimize healing in these conditions remains limited, particularly in DM1, which is characterized by pronounced osteogenic impairment. For this reason, researchers from the Center for the Study of Venoms and Venomous Animals (CEVAP, São Paulo State University, UNESP, Botucatu, Brazil) developed a heterologous fibrin biopolymer (HFB) derived from a fraction of *Crotalus durissus terrificus* snake venom combined with a fibrinogen-rich cryoprecipitate obtained from buffalo (*Bubalus bubalis*) blood [23,24].

Preclinical and clinical studies have demonstrated its safety and efficacy in treating chronic venous ulcers in humans, with or without DM [25]. HFB exhibits affinity for bone cells [26] and possesses key properties, including biocompatibility, biodegradability, and bioactivity, which are essential for biomaterials used in tissue engineering [27]. Favorable in vivo results have also been reported for its osteoconductive capacity in repairing critical-size calvarial defects [28], suggesting its potential as a biological scaffold that supports cell migration, angiogenesis, and new bone formation. In addition, a previous study demonstrated that HFB effectively suppresses osteoclast activity and reduces alveolar bone resorption, particularly during the early healing period in normoglycemic rats [29].

Therefore, the present study investigated the potential association of HFB with post-extraction alveolar bone healing under STZ-induced diabetic conditions, using the blood clot as a biological control.

## 2. Materials and Methods

### 2.1. Study Design

This study was approved by the Institutional Animal Care and Use Committee of UNIMAR (CIAEP Certificate—01.0218.2014, 25 August 2021), in accordance with the ARRIVE 2.0 guidelines (NC3Rs—Animal Research: Reporting of In Vivo Experiments) [30,31].

The sample size was calculated based on the histomorphometric bone area data reported by Arantes et al. 2015 [32]. Using the reported means and standard deviations, the standardized effect size was estimated (Cohen’s d = 1.28). Assuming a two-tailed independent Student’s *t*-test, α = 0.05, and 80% statistical power, G*Power 3.1 (Heinrich-Heine-Universität Düsseldorf, Düsseldorf, Germany) indicated a minimum required sample size of 8 animals per group. A total of 48 adult male Wistar rats (*Rattus norvegicus*), albino coat, aged 120 days and weighing 370 g, were used in this study.

Animals were housed in polypropylene cages in groups of four under controlled environmental conditions (temperature 24 °C; relative humidity 45–65%) and a 12 h light/dark cycle, with ad libitum access to water and a balanced diet containing 1.4% calcium and 0.8% phosphorus (Nuvilab^®^ CR-1; Nuvital Nutrientes S/A, Curitiba, PR, Brazil). All experiments were conducted using calibrated, blinded assessments.

### 2.2. Induction of DM1 by Streptozotocin and Experimental Groups

Streptozotocin (STZ) induces selective β-cell injury, resulting in marked hyperglycemia and reduced pancreatic insulin production. Residual β-cell function may persist depending on the experimental conditions; therefore, this model is characterized by reduced rather than absent endogenous insulin production [33]. A total of 48 animals received a single intraperitoneal injection of STZ (52 mg/kg; S0130, Sigma-Aldrich, St. Louis, MO, USA) to induce DM1 [34,35]. Seven days after induction, animals were fasted for 12 h, and fasting blood glucose levels were measured using an Accu-Chek^®^ system (Roche Holding AG, Basel, Switzerland). Animals presenting fasting blood glucose levels ≥ 250 mg/dL were considered diabetic and eligible for inclusion in the experimental analyses [36,37,38].

Subsequently, the animals were allocated into two experimental groups: blood clot group (BCG; *n* = 24) and HFB group (HFBG; *n* = 24). Randomization was performed using a computer-generated randomization list (Stata, Version 9.0; StataCorp LLC, College Station, TX, USA).

### 2.3. Tooth Extraction

Surgery was performed 7 days after STZ administration. Animals underwent a 6 h preoperative fasting period, followed by general anesthesia via intramuscular injection of tiletamine and zolazepam hydrochloride (10 mg/kg each; Telazol^®^, Fort Dodge Animal Health, Campinas, SP, Brazil). The right maxillary incisor was luxated and subsequently extracted. In the BCG, the extraction sockets were filled with blood clots, and the gingival margins were closed with 4–0 Vicryl^®^ sutures (Ethicon, São Paulo, SP, Brazil). In the HFBG, the extraction sockets were filled with 40 µL of fraction I and 40 µL of the diluent, followed by the addition of 80 µL of fraction II, which rapidly converted into a dense fibrin network (fibrin clot). In this group, socket closure was achieved solely with the HFB, without sutures.

All animals received a single intramuscular injection of 2.5% enrofloxacin (0.2 mL; Flotril^®^, Schering-Plough, Santo Amaro, SP, Brazil). Postoperative analgesia was provided with dipyrone (0.06 mL/kg; Analgex^®^, Agener União, São Paulo, SP, Brazil) every 12 h for 3 days.

### 2.4. Euthanasia, Samples Distribution, and Processing

**Figure 1:**
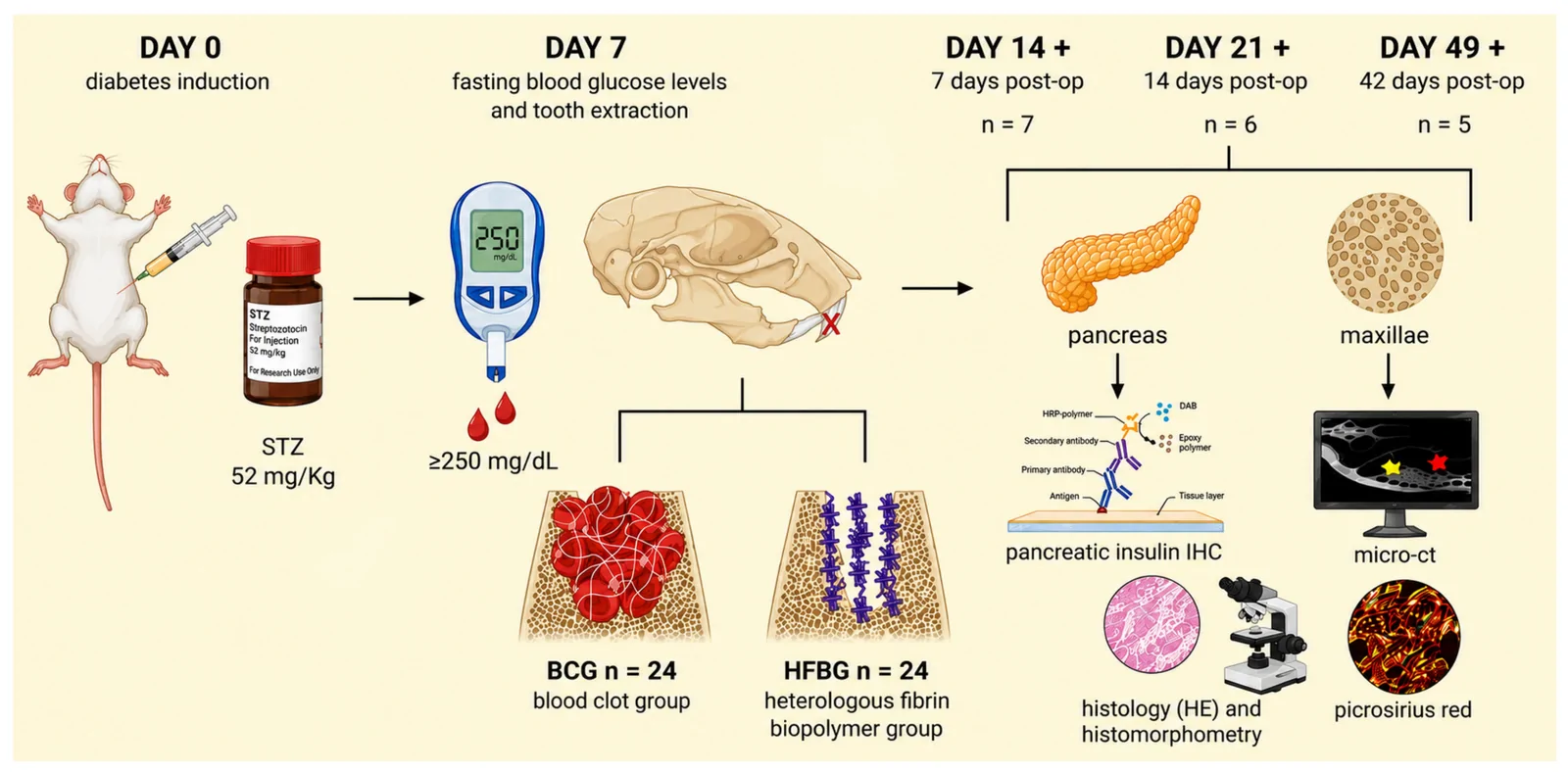
Experimental design and timeline. On day 0, DM1 was induced by STZ. On Day 7, fasting blood glucose was measured, followed by extraction of the right maxillary incisor. Animals were allocated to the blood clot group (BCG) or HFB group (HFBG). Euthanasia was performed at 7, 14, and 42 days post-operatively. The pancreas was collected for insulin immunohistochemistry, and the maxillae were processed for micro-CT, histology (HE), histomorphometry, and picrosirius red analyses.

Fasting blood glucose levels were measured at 7, 14, and 42 days post-extraction, immediately before euthanasia, which was performed by anesthetic overdose via intramuscular administration of tiletamine and zolazepam hydrochloride (30 mg/kg each; Telazol^®^, Fort Dodge Animal Health, Campinas, SP, Brazil), followed by cervical dislocation as a secondary physical method to ensure death. No animals died during the experimental period. Each experimental period initially included eight animals per group (16 animals per time point). Animals presenting fasting blood glucose levels < 250 mg/dL at their respective experimental endpoint were excluded from the statistical analyses according to the predefined glycemic criterion. Six animals met this exclusion criterion (7 days: *n* = 1 (BCG); 14 days: *n* = 2 (1 animal from BCG and 1 from HFBG); 42 days: *n* = 3 (1 animal from BCG and 2 from HFBG), resulting in final sample sizes of *n* = 7, *n* = 6, and *n* = 5 animals per group at the 7-, 14-, and 42-day time points, respectively (Figure 1; Table 1).

The maxillae and pancreas were fixed in 10% neutral-buffered formalin for 48 h, then washed under running water for 24 h. Pancreatic insulin immunohistochemistry (IHC) was used to characterize DM1. Maxillae specimens intended for micro-computed tomography (micro-CT) were stored in 70% ethanol, whereas specimens for histological (HE), histomorphometric, and picrosirius red analysis were decalcified in 4.13% EDTA (pH 7.2) for 20 days. The experimental design and timeline of the study are presented in Figure 1.

### 2.5. Immunohistochemistry

To characterize the DM1 condition through insulin expression, processed pancreatic tissues were embedded in paraffin blocks, sectioned at 4 µm thickness, mounted on silanized glass slides, and subjected to IHC using an anti-insulin antibody (2D11-H5; sc-8033, mouse monoclonal; Santa Cruz Biotechnology, Santa Cruz, CA, USA) and a polymer-based detection system (N-Histofine^®^ Simple Stain^®^ Mouse MAX PO(M); Nichirei Biosciences, Tokyo, Japan), according to the manufacturer’s instructions. From each pancreas, three histological sections were digitized using an Aperio CS2 slide scanner (Leica Biosystems^®^, Nussloch, Germany) at 40× magnification.

Morphometric analysis was performed using the Aperio ScanScope^®^ XT analysis system (Leica Biosystems^®^, Nussloch, Germany) with the Positive Pixel Count v9 algorithm. In this system, insulin immunostaining intensity is color-coded (red, strong; orange, moderate; yellow, weak; blue, negative). The percentage of insulin-positive islets of Langerhans and the distribution of staining intensities in the endocrine pancreas were used to assess overall insulin immunoreactivity.

### 2.6. Micro-CT

The maxillae were scanned using a micro-CT (SkyScan 1174; Bruker^®^, Kontich, Belgium) at 50 kV, 800 µA, and a pixel size of 14 µm. A 0.05 mm Al filter was applied, with a 360° rotation and a 0.7° rotation step. The images were reconstructed with NRecon software (version 1.7; Bruker^®^, Kontich, Belgium) and spatially realigned in the sagittal, transaxial, and coronal planes using DataViewer software (version 1.5.; Bruker^®^, Kontich, Belgium). In the transaxial plane, using CTAn software (version 1.6; Bruker^®^, Kontich, Belgium). The total volumes of the contralateral incisor socket (TVis; mm^3^) and the toothless socket (TVts; mm^3^) were measured. In addition, the following parameters were evaluated, considering the toothless socket as a region of interest (ROI) [39]: socket reduction (SR; %), bone volume (BV; mm^3^), bone volume fraction (BV/TV; %), trabecular number (Tb.N; 1/mm), trabecular thickness (Tb.Th; mm), and trabecular separation (Tb.Sp; mm). The images were reconstructed and spatially realigned in the sagittal, transaxial, and coronal planes; however, quantitative analysis was performed in the transaxial plane using the toothless socket as the region of interest, following the predefined protocol adopted in this study.

### 2.7. Histology (HE) and Histomorphometry

Maxillary tissues were embedded in paraffin blocks and sectioned at 4 µm. Longitudinal serial sections were obtained along the mesiodistal axis of the alveolar socket. The sections were mounted on silanized glass slides, stained with hematoxylin and eosin (HE), and digitized using an Aperio CS2 slide scanner (Leica Biosystems^®^, Nussloch, Germany) at 20× magnification. Qualitative histological evaluation was performed using ImageScope software (version 12.3.3; Leica Biosystems^®^, Nussloch, Germany), considering the type and intensity of the inflammatory response and tissue healing processes, including fibrosis, neovascularization, and bone formation and remodeling.

For histomorphometric analysis, the contralateral intact socket (TAis; mm^2^) and the total area of the toothless socket (TAts; mm^2^) were measured. Within the toothless socket, the following parameters were evaluated [40]: socket reduction (SR; %), bone area fraction (BA/TAts; %), connective tissue area fraction (CT/TAts; %), and inflammatory infiltrate area fraction (Ia/TAts; %). Longitudinal serial sections were obtained along the mesiodistal axis of the alveolar socket. Representative histological images were selected to illustrate the observed healing patterns within each experimental condition and time point, rather than to indicate mirrored or symmetrical sampling sites from the same specimen.

### 2.8. Picrosirius Red

The quantification of birefringent fibers during the alveolar bone healing process was performed on histological sections stained with picrosirius red. Four images per socket were acquired using a linear polarized light microscope (DMRB; Leica Biosystems^®^, Nussloch, Germany) at 5× and 20× magnifications, with standardized light intensity and polarizer angle (90° relative to the light source). This stain allows the evaluation of collagen maturation and organization under polarized light, in which thick, mature collagen fibers exhibit red-orange birefringence, whereas thin, immature fibers appear greenish [41]. The total percentage of birefringent fibers and the distribution of polarization colors were determined using Leica Application Suite X software (version 5.3.3, LAS X; Leica Microsystems^®^, Nussloch, Germany).

### 2.9. Statistical Analysis

Statistical analysis was performed using Statistica 10.0 software (StatSoft Inc., Tulsa, OK, USA). Data normality was assessed using the Shapiro–Wilk test, and homogeneity of variances was evaluated using Levene’s test (*p* > 0.05). For comparisons among experimental periods, a one-way ANOVA followed by Tukey’s post hoc test was used. For comparisons between independent groups (BCG vs. HFBG) within each experimental period, an independent Student’s *t*-test was used. The significance level was set at 5% (*p* < 0.05), and data are presented as mean ± standard deviation. These analyses were intended to address predefined stage-specific comparisons in a terminal experimental design and should be interpreted accordingly.

## 3. Results

### 3.1. Characterization of the DM1 Condition

**Figure 2:**
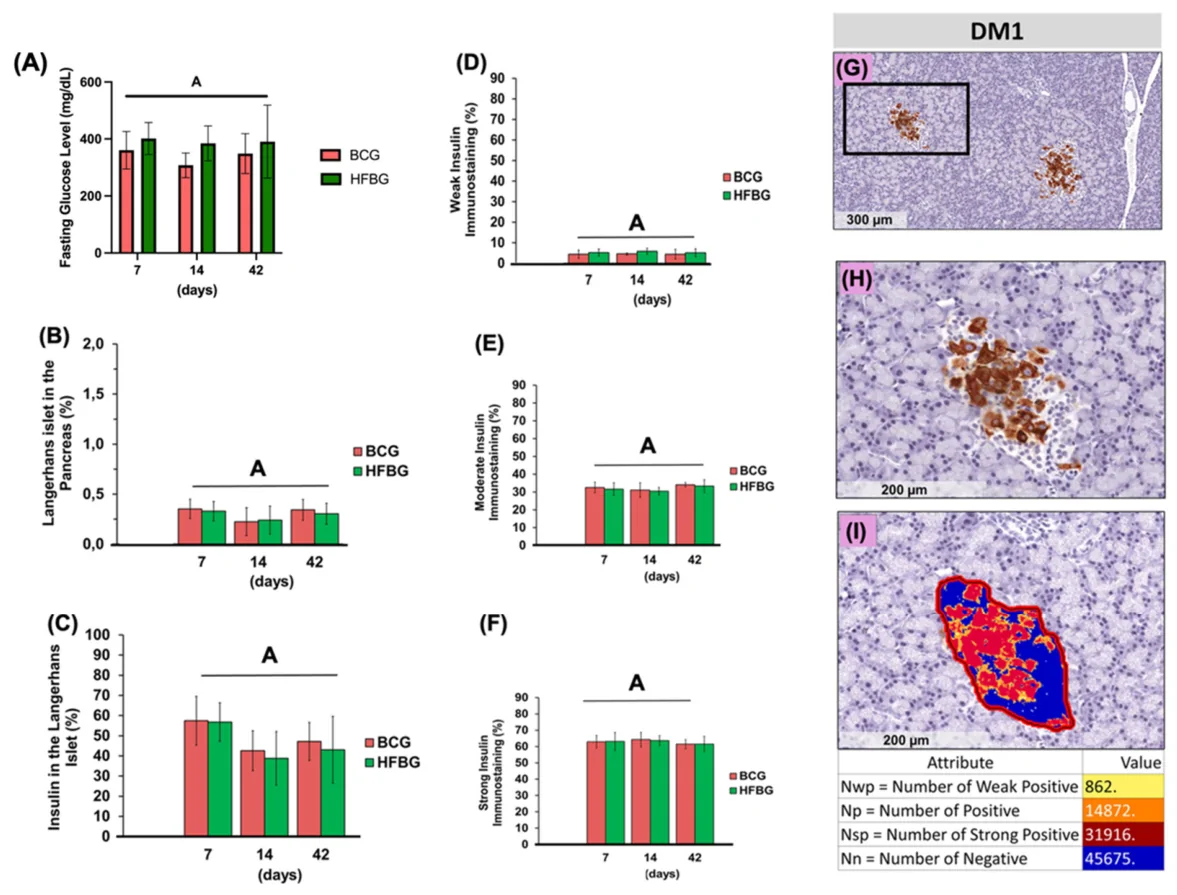
Characterization of DM1. (**A**) Fasting blood glucose levels in the BCG and HFBGs at 7, 14 and 42 days. (**B**) Langerhans islet in the pancreas. (**C**) Insulin in the Langerhans islet. (**D**) Weak insulin immunostaining. (**E**) Moderate insulin immunostaining. (**F**) Strong insulin immunostaining. (**G**,**H**) Reduced size of the islets of Langerhans in the endocrine pancreas. (**I**) Insulin immunostaining intensity algorithm: red, strong; orange, moderate; yellow, weak; blue, negative. No statistically significant differences were found between groups and/or experimental periods, as indicated by the same letter (A) (*p* > 0.05).

Fasting blood glucose levels of animals meeting the predefined glycemic inclusion criterion (≥ 250 mg/dL) at the respective experimental endpoints (7, 14, and 42 days) are presented in Figure 2A. Persistent hyperglycemia was observed at all evaluated time points, with no significant differences between the experimental groups (*p* > 0.05). The islets of Langerhans occupied, on average, 0.30% of the pancreatic area (Figure 2B). Within the islets, insulin immunostaining averaged 47.6% (Figure 2C). A predominance of weak (Figure 2D) and moderate (Figure 2E) insulin expression was observed in β-cells (Figure 2F). Representative images illustrate the reduced size of the islets of Langerhans (Figure 2G,H), and the marker classification algorithm used for immunohistochemical quantification is shown in Figure 2I. No significant differences were observed between the experimental groups for any of these pancreatic parameters (*p* > 0.05).

### 3.2. Micro-CT

**Figure 3:**
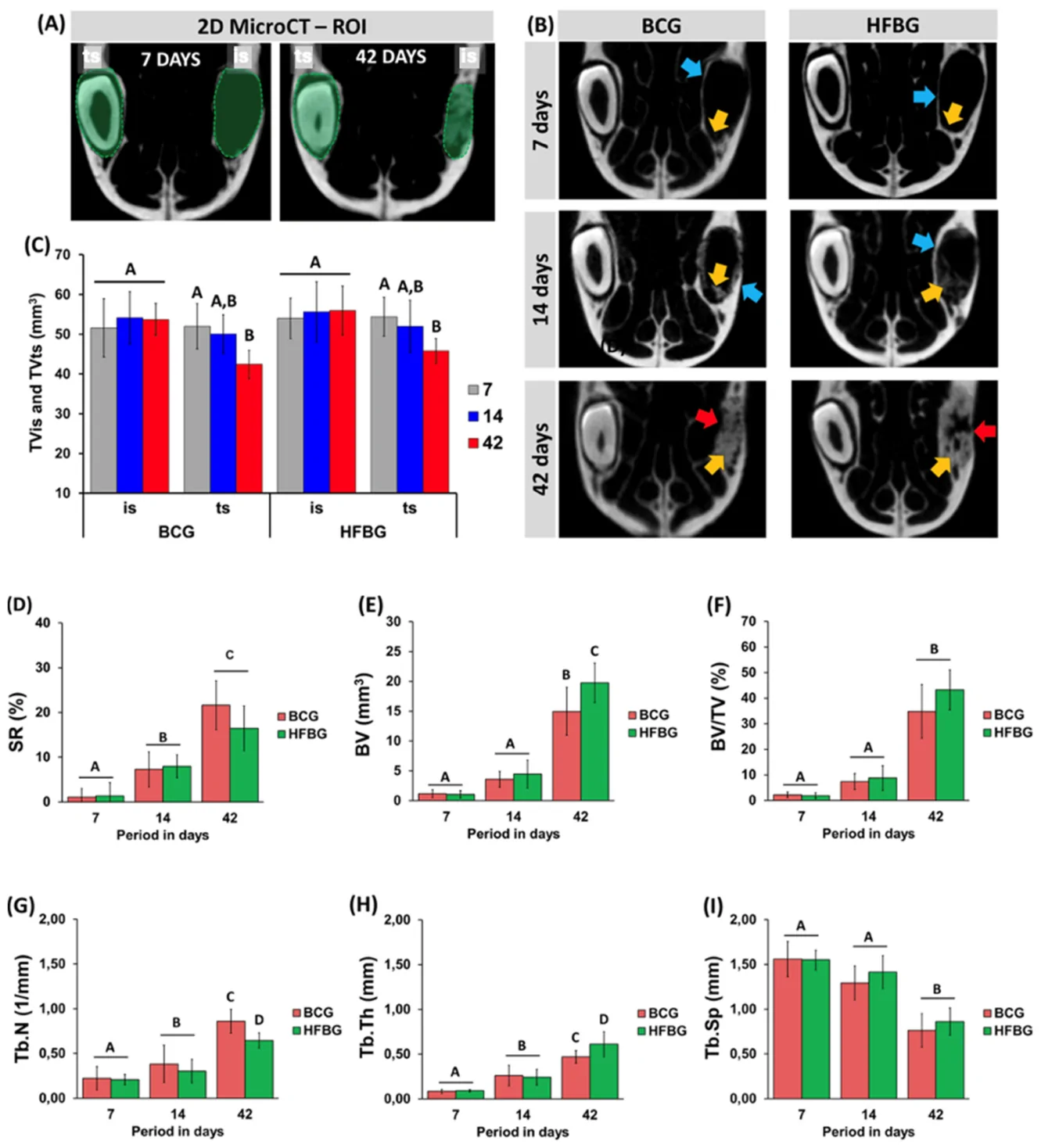
Micro-CT. (**A**) 2D transaxial images showing the region of interest used for volumetric analysis of the toothless socket (ts) and the contralateral incisor socket (is) at 7 and 42 days. (**B**) 2D transaxial images of the medial third socket. At 7 days, the ts-socket shows a well-preserved contour (blue arrow). At 14 days, small radiodense areas appear within the socket (yellow arrow), indicating new bone formation. At 42 days, the ts-socket is reduced, with altered alveolar contour (red arrow) and increased radiodensity (yellow arrow). (**C**) TV, total; TVis, contralateral intact socket; TVts, toothless socket. (**D**) SR, socket reduction. (**E**) BV, bone volume. (**F**) BV/TV, bone volume fraction. (**G**) Tb.N, trabecular number. (**H**) Tb.Th, trabecular thickness. (**I**) Tb.Sp, trabecular separation. Different letters indicate statistically significant differences between groups and/or experimental periods (*p* < 0.05).

Micro-CT analysis showed a reduction in total volume of tissue section (TVts) at 42 days, with an evident alteration in the alveolar contour compared with tissue volume inside the selected region (TVis) (Figure 3A), and bone formation progressing from the alveolar walls toward the socket center (Figure 3B). Volumetric analysis confirmed that TVts was significantly lower than TVis at all time points (Figure 3C; *p* < 0.05). At 42 days, TVts (43.96 mm^3^) was 19.2% lower than TVis (54.42 mm^3^). No differences were observed between BCG and HFBG for TVts or TVis at any period (Figure 3C; *p* > 0.05).

Socket reduction (SR) varied significantly over time in both groups (Figure 3D; *p* < 0.05), with no intergroup differences (Figure 3D; *p* > 0.05). At 42 days, bone volume (BV) was 31.86% higher in the HFBG than in the BCG (Figure 3E; *p* < 0.05). Bone volume fraction (BV/TV) increased significantly at 42 days compared with 7 and 14 days (Figure 3F; *p* < 0.05), with no differences between groups (Figure 3F; *p* > 0.05). The descriptive statistics (n, mean, SD, and 95% confidence interval, *p*-value and effect size) for tissue volume (TV), BV, and BV/TV at the 42-day time point are provided in Supplementary Table S1. Regarding microarchitecture, trabecular number (Tb.N) and trabecular thickness (Tb.Th) varied significantly over time in both groups (Figure 3G,H; *p* < 0.05). At 42 days, BCG exhibited higher Tb.N, whereas HFBG exhibited higher Tb.Th (Figure 3G,H; *p* < 0.05). Trabecular separation (Tb.Sp) decreased significantly at 42 days compared with earlier periods (Figure 3I; *p* < 0.05), with no intergroup differences (Figure 3I; *p* > 0.05).

### 3.3. Histological and Histomorphometric Analysis

**Figure 4:**
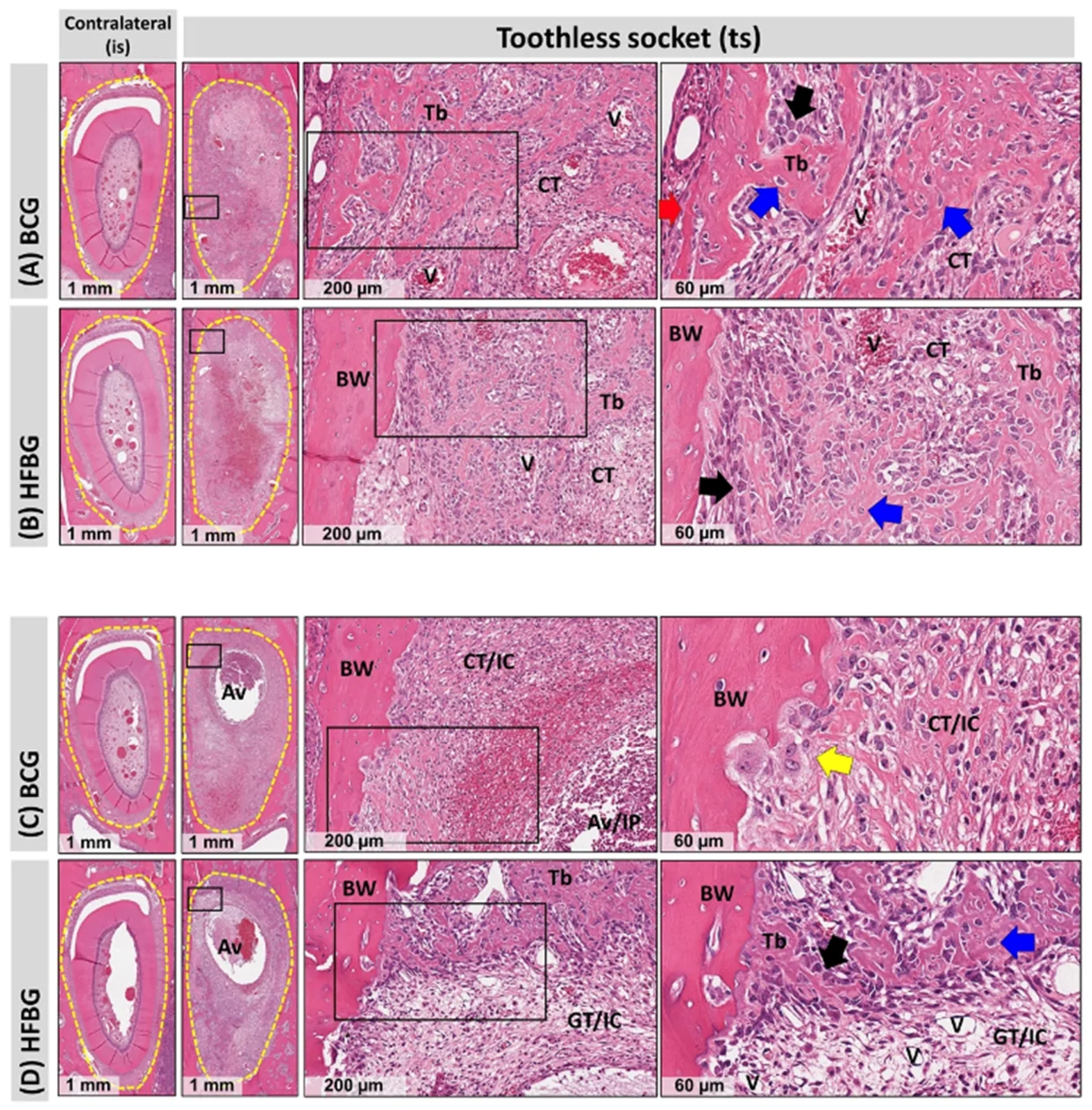
Representative histological images of alveolar bone healing at 7 days. Images were selected to illustrate the main tissue patterns observed in each experimental group and period and are presented for descriptive purposes. (**A**,**C**) BCG. (**B**,**D**) HFBG. Av, alveolitis. AV/IP, inflammatory process. BW, bone wall. GT/CT, granulation/connective tissue. GT/IC, granulation tissue/inflammatory cells. V, blood vessels. Tb, trabecular bone. Blue arrow, osteocytes. Black arrow, osteoblasts. Red arrow, bone wall resorption. Yellow arrow, alveolitis.

**Figure 5:**
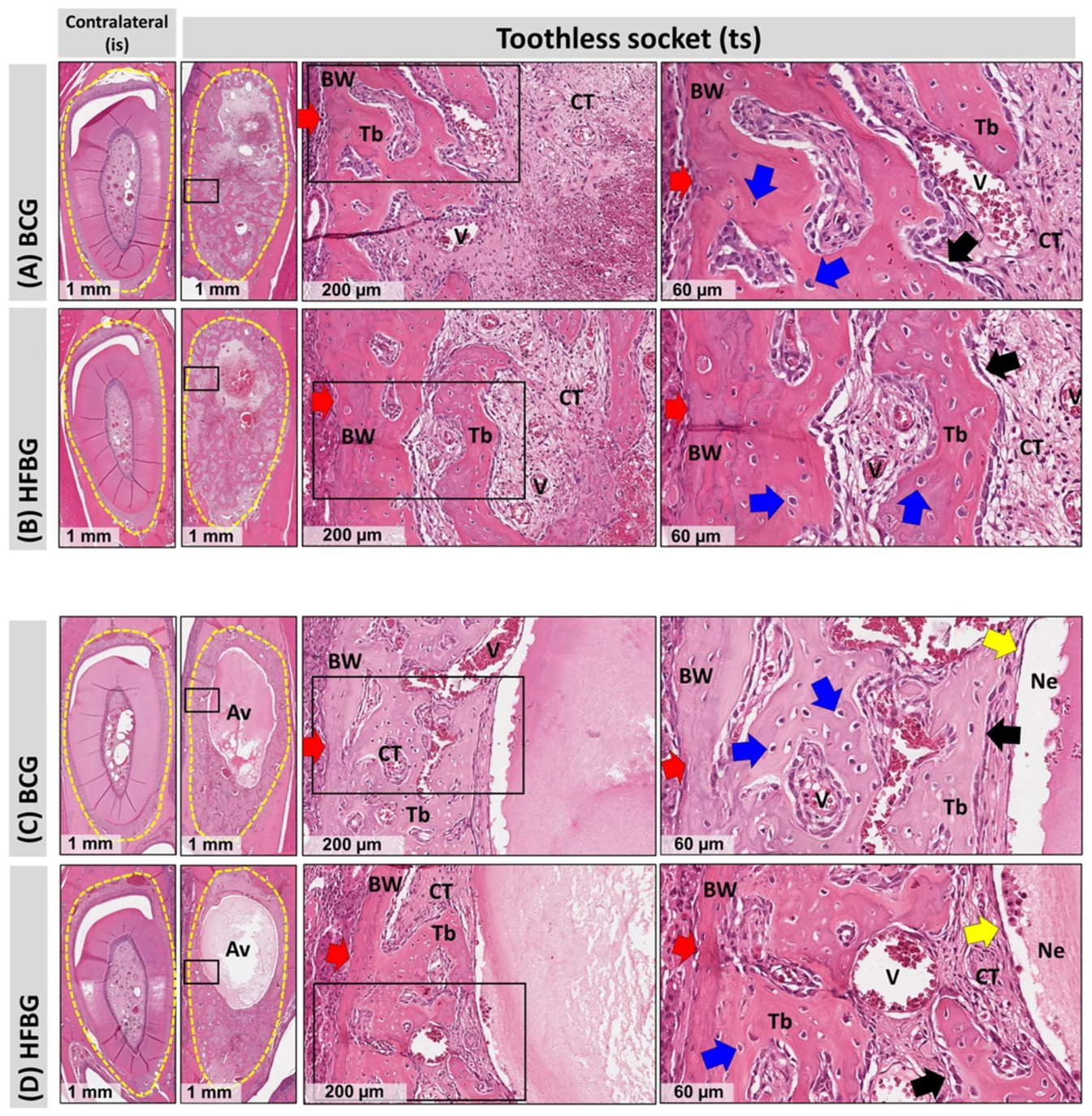
Representative histological images of alveolar bone healing at 14 days. Images were selected to illustrate the main tissue patterns observed in each experimental group and period and are presented for descriptive purposes. (**A**,**C**) BCG group. (**B**,**D**) HFBG. BW, bone wall. CT, connective tissue. Ne, eosinophilic acellular material. V, blood vessels. Tb, trabecular bone. Blue arrow, osteocytes. Black arrow, osteoblasts. Red arrow, bone wall resorption. Yellow arrow, fibrous connective capsule.

**Figure 6:**
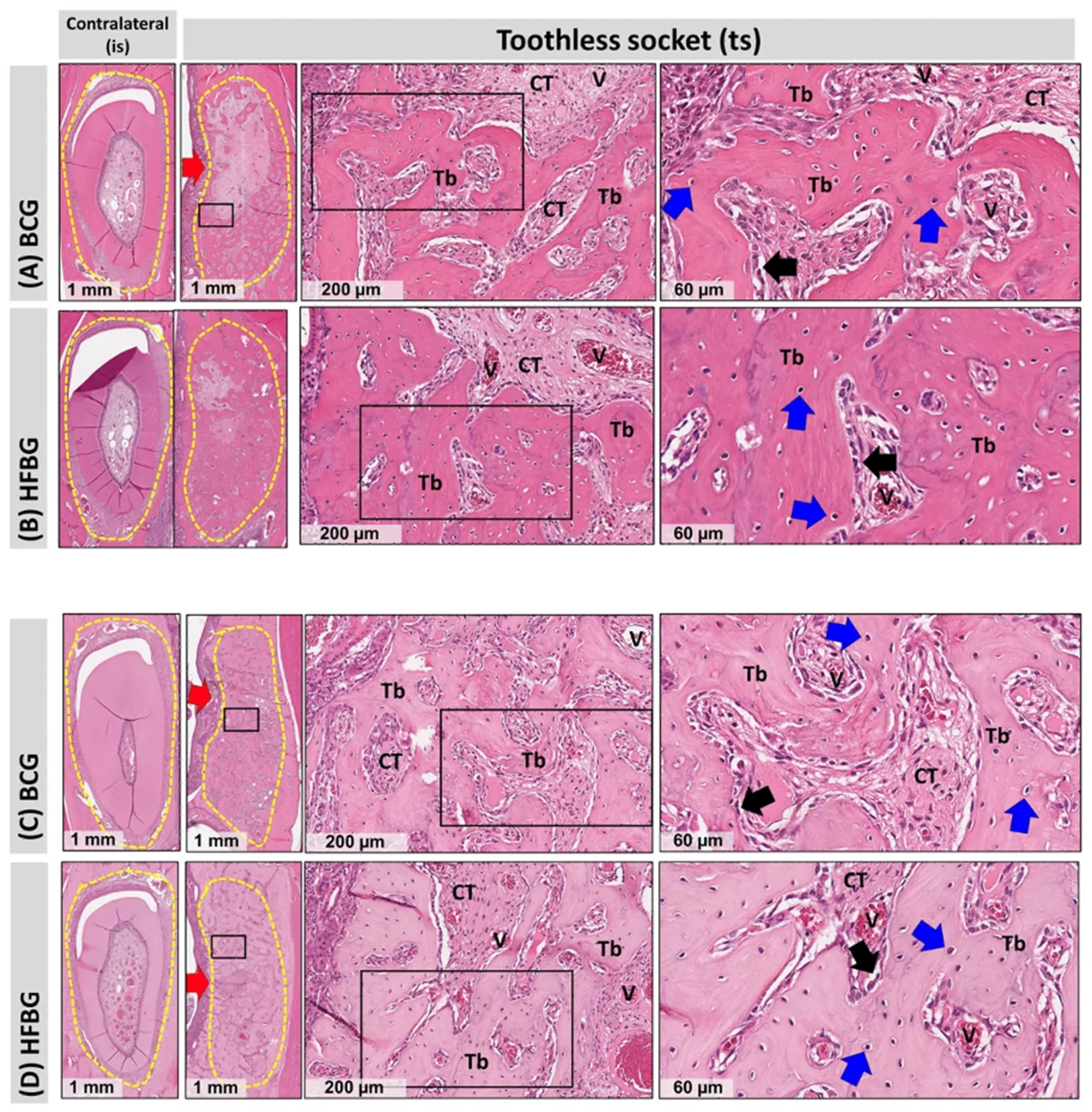
Representative histological images of alveolar bone healing at 42 days. (**A**,**C**) BCG. (**B**,**D**) HFBG. CT, connective tissue. V, blood vessels. Tb, trabecular bone. Blue arrow, osteocytes. Black arrow, osteoblasts. Red arrow, bone wall resorption.

**Figure 7:**
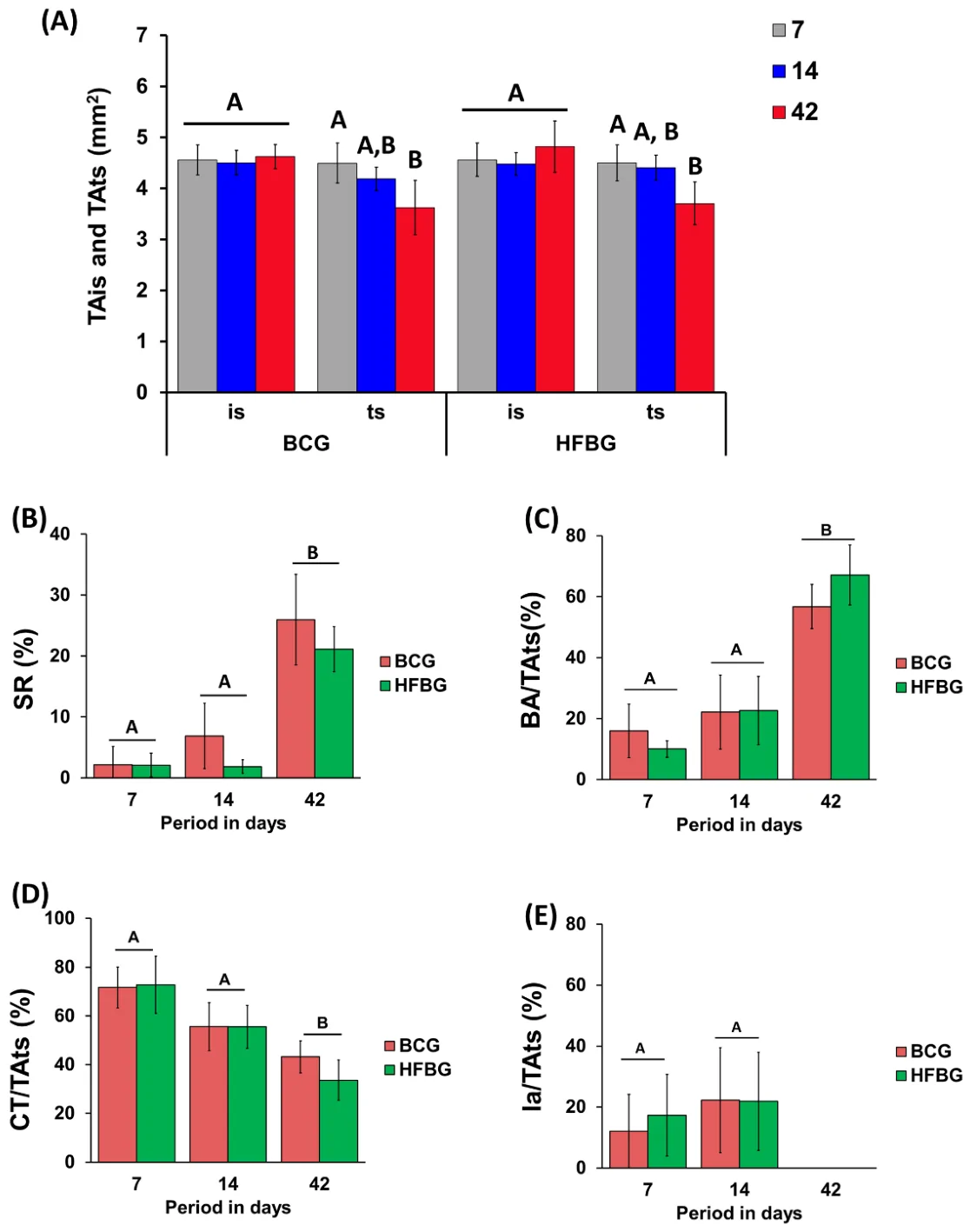
Histomorphometry. (**A**) Tais, contralateral intact socket; TAts, total area of the toothless socket. (**B**) SR, socket reduction. (**C**) BA/TAts, bone area fraction. (**D**) CT/TAts, connective tissue area fraction. (**E**) Ia/TAts, inflammatory infiltrate area fraction. Different letters indicate statistically significant differences between groups and/or experimental periods (*p* < 0.05).

Histological analysis is presented in Figure 4, Figure 5 and Figure 6, while histomorphometry is shown in Figure 7. The representative histological images were organized only to facilitate visualization of the tissue patterns observed during healing and were not intended to indicate paired symmetrical sampling sites.

At 7 days (Figure 4), the toothless alveolar socket showed small areas of inflammatory infiltrate associated with immature bone formation adjacent to the alveolar bone wall. Immature trabecular bone containing numerous osteocytes and active osteoblasts lining the bone surface was observed, while the remaining alveolar spaces were filled with granulation tissue or highly vascularized connective tissue (Figure 4A,B). Bone wall resorption was also noted in the BCG (Figure 4A). In the BCG, alveolitis was characterized by an inflammatory infiltrate associated with resorption of the alveolar bone wall (Figure 4C). In contrast, the HFBG exhibited a blood clot, granulation tissue containing inflammatory cells and blood vessels, trabecular bone formation adjacent to the alveolar bone wall, and active osteoblasts lining the bone surface (Figure 4D).

At 14 days (Figure 5), the ts-socket contours exhibited fewer alterations than the is-socket. Bone wall resorption was evident, accompanied by trabecular bone formation containing dispersed osteocytes and lining osteoblasts. The remaining alveolar spaces were filled with highly vascularized connective tissue (Figure 5A,B), trabecular bone with osteocytes and osteoblasts was also observed. However, part of the socket was occupied by eosinophilic acellular material, which may represent residual biomaterial. Bone wall resorption was also observed in the specimens shown in Figure 5C,D.

Area of alveolitis persisted for up to 14 days, characterized by an inflammatory infiltrate and eosinophilic acellular material (Figure 4C and Figure 5C,D). In contrast, at 42 days, the alveolitis disappears (Figure 6A–D).

At 42 days (Figure 6), ts-socket contours showed higher alterations than the is-socket. Intense resorption of the alveolar bone walls and ongoing bone remodeling resulted in a marked reduction in the alveolar socket. In all cases, complete resorption of the vestibular and palatal bone walls was observed, leading to a reduced alveolar space predominantly occupied by mature trabecular bone containing osteocytes and lining osteoblasts, interspersed with highly vascularized connective tissue. The HFBG exhibited a more mature, compact, and organized bone structure, whereas the BCG presented bone tissue with a more trabecular and immature architecture (Figure 6A–D).

Histomorphometric analysis showed that contralateral intact socket (Tais) remained similar across all experimental periods and between groups (mean 4.6 mm^2^; Figure 7A; *p* > 0.05). In contrast, total area of the toothless socket (Tats) decreased significantly over time, with lower values at 42 days (3.7 mm^2^) than at 7 days (4.5 mm^2^) (Figure 7A; *p* < 0.05). However, no significant differences were observed between the BCG and HFBGs at any time point (Figure 7A; *p* > 0.05). SR was 26.0% in BCG and 21.1% in HFBG, with no significant difference between groups (Figure 7B; *p* > 0.05). The bone area fraction (BA/Tats) was lower at 7 days (13%) and 14 days (22.4%) and increased significantly at 42 days (62%) (Figure 7C; *p* < 0.05), with a reduction in connective tissue area fraction (CT/Tats) (Figure 7D; *p* < 0.05). No differences were observed for inflammatory infiltrate area fraction (Ia/Tats) over time or between groups (Figure 7E; *p* > 0.05).

### 3.4. Picrosirius Red (Collagen Fibers)

**Figure 8:**
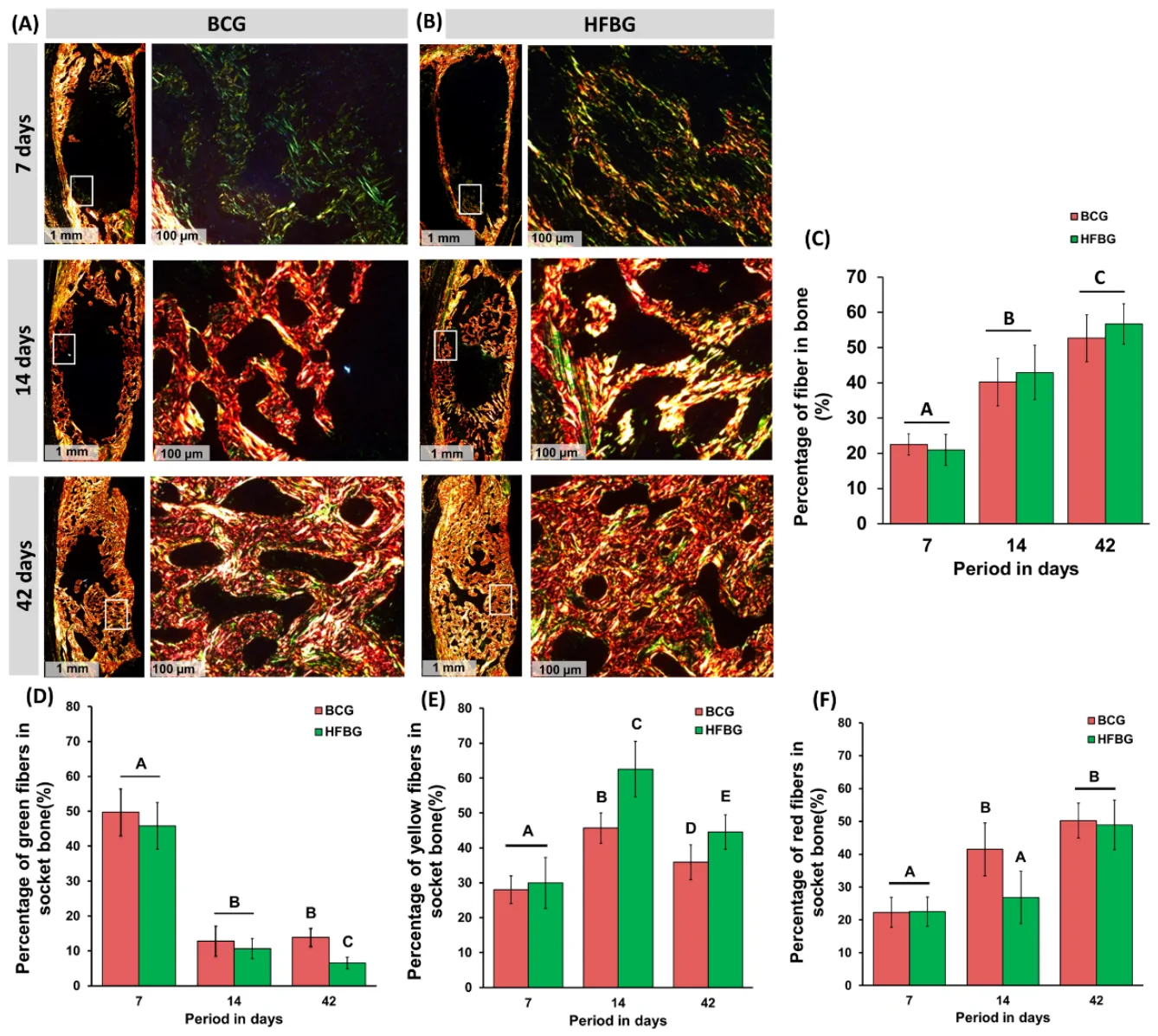
Representative polarized-light images are shown together with the corresponding semi-quantitative analysis of collagen fiber distribution. The quantitative data directly refer to the same experimental groups and time points illustrated in the image panels. (**A**,**B**) Representative polarized-light images showing thin collagen fibers (green), intermediate (yellow), and thick collagen fibers (red), at 7, 14 and 42 days. (**C**) Percentage of fiber in bone. (**D**) Percentage of green fibers in the socket bone. (**E**) Percentage of yellow fibers in the socket bone. (**F**) Percentage of red fibers in the socket bone. Different letters indicate statistically significant differences between groups and/or experimental periods (*p* < 0.05).

Representative polarized-light images showing thin (green), intermediate (yellow), and thick (red) collagen fibers at different time points are presented in Figure 8A,B. In both groups, the total collagen content in newly formed bone increased significantly over time, from 21.7 ± 3.7 at 7 days to 54.7 ± 6.2 at 42 days (Figure 8C; *p* < 0.05).

Thin collagen fibers predominated at 7 days in both groups, with no intergroup differences (Figure 8D; *p* > 0.05), followed by a significant reduction at 14 and 42 days (Figure 8D; *p* < 0.05). At 42 days, HFBG showed the lowest percentage of thin fibers, whereas BCG maintained similar values from 14 to 42 days (Figure 8D; *p* < 0.05). Intermediate collagen fibers increased significantly at 14 days in both groups, particularly in HFBG (Figure 8E; *p* < 0.05), and decreased at 42 days, remaining higher than at 7 days and without intergroup differences (Figure 8E; *p* > 0.05). Thick collagen fibers increased progressively over time, with significantly higher values at 14 and 42 days compared with 7 days (Figure 8F; *p* < 0.05). At 42 days, both groups exhibited the highest percentages of thick fibers, with no differences between BCG and HFBG (Figure 8F; *p* > 0.05).

To support translational interpretation, the present DM1 findings were compared with our previous study in normoglycemic rats [29], which used the same experimental and HFB protocols. The comparative analysis is summarized in Table 2.

**Figure 9:**
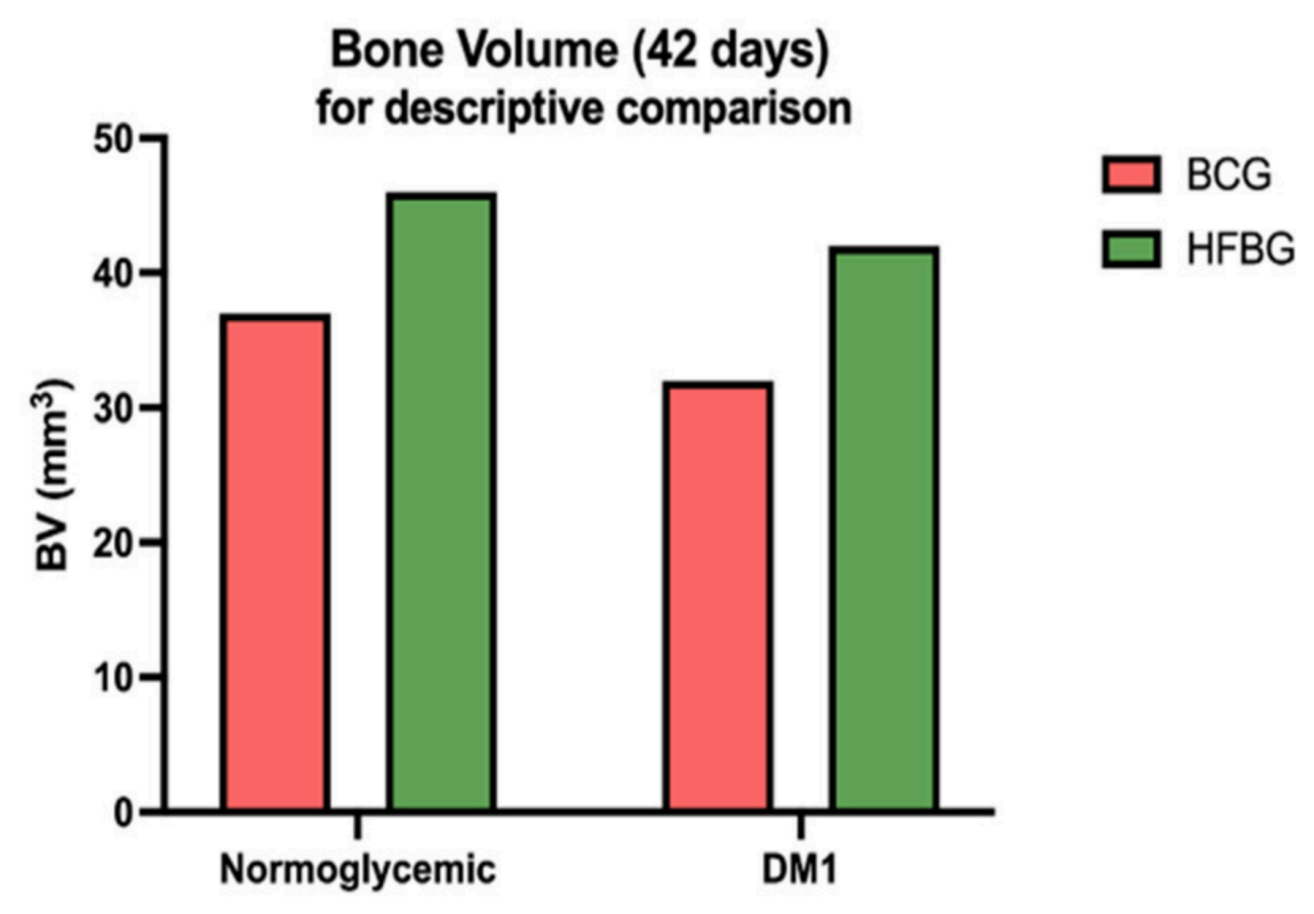
Bone volume (BV) at 42 days in sockets filled with blood clot or HFB in normoglycemic and STZ-induced DM1 rats. Normoglycemic data were extracted from a previously published study using the same surgical and biomaterial protocol [29] and are shown for descriptive comparison only.

For descriptive purposes, BV values at 42 days were qualitatively compared with those reported in our previous normoglycemic study using the same HFB protocol [29] (Figure 9). Although similar trends were observed in both studies, these data originated from independent experiments and were not statistically compared. Therefore, this comparison should be interpreted as descriptive and does not demonstrate preservation of the HFB effect under diabetic conditions.

## 4. Discussion

The present study evaluated the effect of HFB on post-extraction alveolar bone healing in DM1. The main findings demonstrate that although early healing was characterized by inflammatory changes and, in some cases, persistent alveolitis for up to 14 days, HFB use was associated with improved bone healing at later stages. At 42 days, HFB-treated sockets exhibited higher bone volume and trabecular thickness, as well as greater collagen fiber maturation, than the BCG control. These results indicate that HFB does not prevent the early inflammatory alterations induced by DM1 but contributes to improved socket repair during the late healing phase.

The impaired alveolar bone healing observed in this study is consistent with the well-documented effects of DM1 on bone metabolism [14,15]. In this experimental model, DM1 was confirmed by sustained hyperglycemia and reduced insulin immunostaining in pancreatic β-cells. Histological findings at early time points, including inflammatory infiltrate, immature bone formation, and alveolitis, corroborate prior evidence of increased susceptibility to bone loss and delayed tissue repair in diabetic conditions [42].

The variability in fasting blood glucose levels observed at 14 and 42 days is a recognized characteristic of the STZ-induced diabetes model and likely reflects inter-animal differences in susceptibility to STZ and residual pancreatic β-cell function. Despite this variability, all animals met the predefined inclusion criterion (fasting blood glucose ≥ 250 mg/dL) at study entry and reduced pancreatic insulin immunostaining further confirmed the diabetic phenotype.

An interesting finding of this study was the identification of two distinct histological patterns during the early stages of healing. While some specimens exhibited immature trabecular bone formation adjacent to the alveolar walls, with active osteoblasts and vascularized connective tissue, others showed persistent alveolitis characterized by inflammatory infiltrate, necrotic tissue, and resorption of the alveolar bone wall.

In addition to the persistent inflammatory state, the deleterious effects of DM1 on bone repair involve multiple molecular pathways linked to chronic hyperglycemia and insulin deficiency. Experimental studies have shown that the diabetic environment impairs osteoblast differentiation and activity, reducing the expression of osteogenic markers such as RUNX2, osteocalcin, and alkaline phosphatase, thereby compromising extracellular matrix deposition and mineralization [6,7,8]. Sustained hyperglycemia also promotes the accumulation of advanced glycation end products (AGEs), which alter collagen organization and increase matrix stiffness and fragility, contributing to inferior bone quality [12,43,44,45]. Furthermore, DM1 is associated with impaired angiogenesis and a persistent pro-inflammatory microenvironment characterized by increased levels of cytokines such as TNF-α, IL-1β, and IL-6, which may stimulate osteoclastogenesis and inhibit osteoblastic activity, ultimately prolonging the inflammatory phase of healing [14,42]. These mechanisms may explain the delayed tissue maturation, persistent alveolitis, and bone wall resorption observed during the early stages of alveolar healing in the present study.

Despite an unfavorable inflammatory environment during early healing, HFB application showed significant benefits during the remodeling phase, as evidenced by higher BV and Tb.Th at 42 days. However, the BCG exhibited higher Tb.N, indicating the blood clot’s intrinsic osteogenic potential for bone repair, particularly in minor bone defects [43]. Although the blood clot represents the initial biological matrix for alveolar healing, it may become unstable in diabetic environments characterized by prolonged inflammation and impaired vascularization.

Histological and histomorphometric findings corroborated the micro-CT results. At 42 days, both groups exhibited marked reduction in the alveolar socket due to intense resorption of the vestibular and palatal bone walls and ongoing remodeling. However, HFB-treated sockets were predominantly occupied by more organized and compact trabecular bone, whereas the BCG retained features of a more immature bone architecture. Histomorphometric analysis confirmed a significant increase in bone area fraction and a concomitant reduction in connective tissue area over time, reinforcing the progressive replacement of soft tissue by mineralized matrix. The absence of significant differences in inflammatory infiltrate area at later stages suggests that the beneficial effects of HFB are more closely related to modulation of bone formation and matrix organization than to direct suppression of inflammation.

Previous preclinical evidence in normoglycemic rats demonstrated that HFB suppresses early osteoclast activity, reduces inflammation, and preserves alveolar ridge dimensions, thereby increasing bone volume and reducing socket resorption [29]. In contrast, under DM1 conditions, the present study demonstrated that HFB primarily improved late-stage healing, as evidenced by increased bone volume, trabecular thickness, and collagen maturation at 42 days. Although DM1 reduced overall BV compared with normoglycemic rats, HFB maintained a consistent relative gain over blood clot in both models, suggesting that its osteogenic effect persists even under severe metabolic impairment. These findings suggest that the biological effects of HFB are highly dependent on the metabolic microenvironment in which healing occurs.

Conversely, in the diabetic microenvironment characterized by persistent inflammation, impaired angiogenesis, collagen disorganization, and reduced osteoblastic function, the role of HFB appears to shift toward assuring a bioactive scaffold that sustains cellular organization and supports tissue repair during the remodeling phase. In this context, the fibrin matrix may help stabilize the healing clot, preserve a provisional extracellular matrix, and maintain a reparative niche favorable for osteogenesis and neovascularization despite chronic metabolic impairment. Although these mechanisms remain hypothetical, the present findings suggest that the behavior of fibrin-based biomaterials may be influenced by the host metabolic condition.

The biological effects of HFB observed in this study may be explained by its ability to form a dense, organized fibrin network that provides structural stability to the alveolar socket while supporting cell adhesion, migration, and proliferation, particularly of osteogenic and endothelial cells [25,27]. In addition to its mechanical role, fibrin-based biomaterials are recognized as provisional extracellular matrices capable of retaining and gradually releasing endogenous growth factors involved in tissue repair, including TGF-β, VEGF, and PDGF. These mediators play important roles in osteoblast recruitment and differentiation, collagen synthesis, neovascularization, and extracellular matrix deposition [44,45].

Therefore, even under metabolically compromised conditions such as DM1, the organized fibrin network provided by HFB may help maintain a more favorable reparative microenvironment, supporting collagen maturation and the formation of thicker, more organized trabeculae, as observed in the present study. Given that DM1 is associated with impaired angiogenesis and persistent inflammatory signaling, the ability of HFB to stabilize the healing clot and potentially concentrate bioactive mediators may help explain its beneficial effects in the late stages of alveolar repair. Nevertheless, these mechanisms remain hypothetical and should be confirmed by future molecular, immunohistochemical, and in vitro investigations.

Collagen is directly associated with bone quality because it plays a central role in matrix organization and mineralization [46]. Therefore, collagen fiber analysis using picrosirius red staining provided additional evidence of improved tissue quality in HFB-treated sockets. The progressive increase in total collagen content over time, together with the transition from thin to thick collagen fibers, reflects extracellular matrix maturation during alveolar bone healing [47,48,49]. Notably, the lower proportion of thin collagen fibers observed in the HFB group at 42 days indicates more advanced collagen organization compared with the BCG control.

From a translational perspective, HFB’s ability to sustain effective bone repair under unfavorable conditions is of considerable clinical interest. In this context, HFB appears to act primarily as a bioactive scaffold that maintains the reparative microenvironment throughout the healing process rather than functioning as a direct anti-inflammatory agent. This mechanism is consistent with a previous study demonstrating the osteoconductive properties of HFB in a rat calvarial bone model [28]. Clinically, platelet-rich fibrin (PRF) has demonstrated beneficial effects on socket healing in patients with DM [19,20]. However, its preparation requires collecting autologous blood, which may be uncomfortable for patients. In this context, HFB emerges as a promising alternative biomaterial that provides structural and biological support for healing without requiring blood harvesting.

Importantly, the future prospects of fibrin-based biopolymers are supported by evidence from the treatment of chronic venous ulcers, with a clinical study having successfully completed Phase II evaluation by the Brazilian National Health Regulatory Agency (ANVISA) [50]. These findings support the continued investigation of HFB for post-extraction alveolar bone healing. However, given the limitations of the continuously erupting rat incisor model, further validation in preclinical models and well-designed clinical studies is required before translation to human applications.

Some limitations of this study should be acknowledged. First, we chose not to include a healthy non-diabetic control group because our group recently published a study using the same HFB methodology [29], primarily for ethical reasons related to the 3Rs principle (Replacement, Reduction, and Refinement). In addition, molecular, physicochemical, and structural analyses of HFB were not performed in this experimental design. Furthermore, it should be noted that no additional quantitative analyses oriented by the alveolus were performed in the sagittal or coronal planes. Such complementary assessments could provide further details in future studies but were not feasible in the present analysis.

Another limitation of this study is that socket closure was not identical between groups. The BCG received conventional sutures, whereas the HFBG relied on HFB for socket closure. Consequently, the observed differences cannot be attributed exclusively to the biomaterial itself, since the closure technique may also have influenced the healing process, particularly with respect to thrombus stability and the early inflammatory response. Therefore, the interpretation of the findings should consider the potential contribution of both the biomaterial and the closure method.

In addition, although the group × time interaction was not formally tested, comparisons performed separately at each experimental time point consistently indicated that the beneficial effects of HFB became evident only at the 42-day endpoint. Therefore, the apparent late-stage effect of HFB should be interpreted within the context of the current statistical design. Furthermore, because animals with fasting glucose levels below 250 mg/dL at the endpoint were excluded, the sample size at 42 days (*n* = 5 per group) was lower than the number indicated by the a priori sample size calculation (8 animals per group). Accordingly, this reduction should be considered when interpreting the findings at this time point.

Furthermore, inflammatory cytokines and growth factors were not evaluated. Future studies should investigate markers such as TNF-α, IL-1β, IL-6, VEGF, and TGF-β to better elucidate the molecular pathways involved in the reparative effects of HFB [51,52,53,54]. Therefore, the present findings should be interpreted cautiously. Moreover, although the STZ-induced DM1 model is well established, further clinical studies are necessary to clarify the potential of HFB within the complex human bone metabolism associated with DM1. In summary, this study demonstrates that HFB was associated with favorable changes in selected late-stage parameters of post-extraction alveolar bone healing in an STZ-induced diabetic model. These findings support the potential of HFB as a bioactive scaffold for alveolar bone healing under metabolically compromised conditions.

## 5. Conclusions

HFB was associated with favorable changes in selected late-stage parameters of post-extraction alveolar bone healing in an STZ-induced diabetic model. Although early healing was characterized by inflammatory changes and, in some cases, persistent alveolitis up to 14 days, the use of HFB was associated with improved bone healing at later stages. At 42 days, HFB-treated sockets exhibited higher bone volume, trabecular thickness, and collagen maturation than BCG control sockets.

## Figures and Tables

**Table 1:** Blood glucose levels (mg/dL) at the experimental endpoints (7, 14, and 42 days) in the BCG and HFB groups. Data are presented as mean ± standard deviation (SD) and range (minimum–maximum). Six animals exhibited fasting blood glucose levels < 250 mg/dL at their respective experimental endpoints and were excluded from the statistical analyses according to the predefined glycemic criterion (7 days: *n* = 1; 14 days: *n* = 2; 42 days: *n* = 3).

Time	Group	*n*	Blood Glucose (mg/dL)
7 days	BCG	7	358 ± 71 (276–468)
	HFBG	7	402 ± 51 (342–481)
14 days	BCG	6	308 ± 42 (260–367)
	HFBG	6	385 ± 66 (270–436)
42 days	BCG	5	349 ± 80 (263–448)
	HFBG	5	391 ± 133 (280–589)

**Table 2:** Summary of quantitative outcomes across time points under DM1 conditions and comparative interpretation with normoglycemic rats. The comparison is descriptive only, because the studies were independent and were not directly compared statistically. HFB showed a pattern broadly consistent with that previously observed under normoglycemic conditions, particularly for selected late-stage parameters.

Parameter	Normoglycemic Rats	STZ-Induced DM1	Comparative Interpretation
Healing periods	7, 14, and 42 days	7, 14, and 42 days	Same healing phases analyzed
BV	Higher BV in HFB group compared with blood clot at 42 days	BV 31.86% higher in HFB compared with blood clot at 42 days	HFB improves bone formation in both models
BV/TV	No significant difference between groups	No significant difference between groups	Similar volumetric bone fraction outcomes
Tb.N	Increased during healing with no major intergroup differences	Higher Tb.N observed in blood clot group at 42 days	DM1 may influence trabecular pattern
Tb.Th	Increased over time during bone maturation	Higher Tb.Th observed in HFB group at 42 days	HFB promotes thicker trabeculae
Tb.Sp	Progressive decrease during healing	Decreased at late healing stages	Similar remodeling pattern
Socket reduction	Lower socket reduction in HFB compared with clot	No significant difference between groups	Dimensional preservation appears attenuated in DM1
Early inflammatory response	Reduced inflammatory infiltrate in HFB group	Persistent inflammation and occasional alveolitis up to 14 days	DM1 delays early healing events
Histological bone organization	Progressive bone maturation and preservation of alveolar ridge	More compact and organized bone structure in HFB group at 42 days	HFB improves late-stage bone repair
Collagen maturation	Progressive increase in thick collagen fibers	Reduced thin fibers and increased collagen maturation in HFB group	Similar matrix maturation trend

BV, bone volume; BV/TV, bone volume fraction; Tb.N, trabecular number; Tb.Th, trabecular thickness; Tb.Sp, trabecular separation.

## Data Availability

The data presented in this study are available on request from the corresponding author.

## Supplementary Materials

The following supporting information can be downloaded at https://www.mdpi.com/article/10.3390/jfb17080380/s1, Table S1. Descriptive statistics (n, mean, SD, and 95% confidence interval, *p*-value, and effect size) for tissue volume (TV), bone volume (BV), and bone volume fraction (BV/TV) at 42 days.
