# Supplementary material for: Heterologous Fibrin Biopolymer for Post-Extraction Alveolar Bone Healing in Streptozotocin-Induced Diabetic Rats
Source: J Funct Biomater. 2026 Aug 3;17(8):380. doi: 10.3390/jfb17080380 (PMC13514214; doi:10.3390/jfb17080380)
Supplement: Supplementary file 1 [file jfb-17-00380-s001.zip › jfb-4409648-supplementary.pdf]

Table S1. Descriptive statistics (n, mean, SD, and 95% confidence interval, p-value, and effect size) for tissue volume (TV), bone volume (BV), and bone volume fraction (BV/TV) at 42 days.

| **Parameter** | **Group** | ***n*** | **Mean ± SD** | **95% CI** | ***p*-value** | **Hedges' g** |
|---|---|---|---|---|---|---|
| **TV ($mm^3$)** | BCG | 5 | 43.31 ± 3.52 | 38.94–47.68 | | |
| | HFBG | 5 | 44.27 ± 3.68 | 39.70–48.84 | 0.68 | 0.24 |
| **BV ($mm^3$)** | BCG | 5 | 14.88 ± 3.00 | 11.16–18.60 | | |
| | HFBG | 5 | 19.62 ± 2.46 | 16.57–22.67 | 0.023 | 1.56 |
| **BV/TV (%)** | BCG | 5 | 34.36 ± 8.08 | 24.33–44.39 | | |
| | HFBG | 5 | 44.33 ± 7.92 | 34.50–54.16 | 0.09 | 1.13 |
